# Clinical and histopathological characteristics of primary focal segmental glomerulosclerosis in Turkish adults

**DOI:** 10.1038/s41598-024-57305-6

**Published:** 2024-03-21

**Authors:** Ilhan Kurultak, Ozkan Gungor, Savas Ozturk, Ahmet Burak Dirim, Necmi Eren, Ezgi Yenigün, Elbis Ahbab Dal, Mevlut Tamer Dincer, Feyza Bora, Suat Akgur, Abdullah Sumnu, Belda Dursun, Savas Sipahi, Hakki Cetinkaya, Idris Sahin, Garip Sahin, Murvet Yilmaz, Bulent Vatansever, Emre Aydın, Memnune Sena Ulu, Ali Gundogdu, Sedat Ustundag, Hayriye Sayarlioglu, Gizem Kumru, Omer C. Elcioglu, Zeki Aydın, Nedim Yılmaz Selcuk, Ceren Onal Guclu, Meric Oruc, Mehmet Kucuk, Nimet Aktas, Ulver Derici, Gultekin Suleymanlar

**Affiliations:** 1 Faculty of Medicine, Department of Internal Medicine, Division of Nephrology, Trakya University, 22030 Edirne, Turkey; 2 Faculty of Medicine, Department of Internal Medicine, Division of Nephrology, Sutcu Imam University, Kahramanmaras, Turkey; 3 Faculty of Medicine, Department of Internal Medicine, Division of Nephrology, Istanbul University, Istanbul, Turkey; 4 Faculty of Medicine, Department of Internal Medicine, Division of Nephrology, Kocaeli University, Kocaeli, Turkey; 5 Department of Internal Medicine, Division of Nephrology, Ankara Numune Training and Research Hospital, Ankara, Turkey; 6 Department of Internal Medicine, Division of Nephrology, Health Science University, Istanbul Hamidiye Sisli Etfal Training and Research Hospital, Istanbul, Turkey; 7 Faculty of Medicine, Department of Internal Medicine, Division of Nephrology, Istanbul University-Cerrahpasa, Istanbul, Turkey; 8 Faculty of Medicine, Department of Internal Medicine, Division of Nephrology, Akdeniz University, Antalya, Turkey; 9 Department of Internal Medicine, Division of Nephrology, Kutahya Health Science University, Evliya Celebi Yuksek Ihtisas Training and Research Hospital, Kutahya, Turkey; 10 Faculty of Medicine, Department of Internal Medicine, Division of Nephrology, Medipol University, Istanbul, Turkey; 11 Faculty of Medicine, Department of InternalMedicine, Division of Nephrology, Pamukkale University, Denizli, Turkey; 12 Faculty of Medicine, Department of Internal Medicine, Division of Nephrology, Sakarya University, Sakarya, Turkey; 13 Department of Internal Medicine, Division of Nephrology, Sultan Abdulhamid Training and Research Hospital, Istanbul, Turkey; 14 Faculty of Medicine, Department of Internal Medicine, Division of Nephrology, Inonu University, Malatya, Turkey; 15 Faculty of Medicine, Department of Internal Medicine, Division of Nephrology, Eskisehir Osmangazi University, Eskişehir, Turkey; 16 Department of Internal Medicine, Division of Nephrology, Health Science University, Bakırköy Dr. Sadi Konuk Training and Research Hospital, Istanbul, Turkey; 17 Department of Internal Medicine, Division of Nephrology, Izmir Bozyaka Training and Research Hospital, Izmir, Turkey; 18 Faculty of Medicine, Department of Internal Medicine, Division of Nephrology, Dicle University, Diyarbakir, Turkey; 19 Faculty of Medicine, Department of Internal Medicine, Division of Nephrology, Afyon Kocatepe University, Afyon, Turkey; 20 Faculty of Medicine, Department of Internal Medicine, Division of Nephrology, Erciyes University, Kayseri, Turkey; 21 Faculty of Medicine, Department of Internal Medicine, Division of Nephrology, Samsun 19 Mayis University, Samsun, Turkey; 22 Faculty of Medicine, Department of Internal Medicine, Division of Nephrology, Ankara University, Ankara, Turkey; 23 Department of Internal Medicine, Division of Nephrology, Bezmialem Vakif University School of Medicine, Istanbul, Turkey; 24 Department of Internal Medicine, Division of Nephrology, Darıca Farabi Training and Research Hospital, Kocaeli, Turkey; 25 Faculty of Medicine, Department of Internal Medicine, Division of Nephrology, Selcuk University, Konya, Turkey; 26 Faculty of Medicine, Department of Internal Medicine, Division of Nephrology, Hacettepe University, Ankara, Turkey; 27 Department of Internal Medicine, Division of Nephrology, Kartal Lutfi Kirdar City Hospital, Istanbul, Turkey; 28 Department of Internal Medicine, Division of Nephrology, Okmeydanı Training and Research Hospital, Istanbul, Turkey; 29 Department of Internal Medicine, Division of Nephrology, Health Science University, Bursa Yuksek Ihtisas Training and Research Hospital, Bursa, Turkey; 30 Faculty of Medicine, Department of Internal Medicine, Division of Nephrology, Gazi University, Ankara, Turkey

**Keywords:** FSGS, Histopathological features, Nephrotic syndrome, Primary focal segmental glomerulosclerosis, Turkish adults, Nephrology, Kidney diseases

## Abstract

The data regarding primary FSGS (pFSGS) from different parts of the world differ. While the prevalence of pFSGS has been increasing in Western countries like the USA, it follows an inconsistent trend in Europe and Asia and a decreasing trend in Far Eastern countries such as China in the last two decades. There are undetermined factors to explain those national and geographic discrepancies. Herein, we aimed to reveal the current prevalence with clinical and histopathological characteristics of pFSGS in Turkish adults. This study includes the biopsy-proven pFSGS patients data recorded between 2009 and 2019, obtained from the national multicenter primary glomerulonephritis registry system of the Turkish Society of Nephrology Glomerular Diseases (TSN-GOLD) database. 850 of the 3875 primer glomerulonephritis patients(21.9%) have pFSGS. The mean age is 40.5 ± 14.2 and 435 (51.2%) of patients are male. Nephrotic syndrome is the most common biopsy indication (59.2%). 32.6% of patients have hematuria, 15.2% have leukocyturia and 7.8% have both. Serum creatinine, albumin, and proteinuria are 1.0 mg/dL (IQR = 0.7–1.4) mg/dl, 3.4 ± 0.9 g/dl, 3400 mg/day(IQR, 1774–5740), respectively. Females have lower mean arterial pressure (− 2.2 mmHg), higher eGFR (+ 10.0 mL/min/1.73 m^2^), and BMI (+ 1.6 kg/m^2^) than males. Thickened basal membrane(76.6%) and mesangial proliferation (53.5%) on light microscopy are the major findings after segmental sclerosis. IgM (32.7%) and C3 (32.9%) depositions are the most common findings on immunofluorescence microscopy. IgM positivity is related to lower eGFR, serum albumin, and higher proteinuria. The prevalence of pFSGS is stable although slightly increasing in Turkish adults. The characteristics of the patients are similar to those seen in Western countries.

## Introduction

Primary focal segmental glomerulosclerosis is the most common cause of the end-stage renal disease (ESRD) among all primary glomerular diseases. The glomerulosclerosis affecting only a part of capillary tuft (segmental), at least one glomerulus (focal) in the glomeruli on light microscopy is the major pathological lesion. Etiopathogenetic approach to FSGS can be classified as primary, genetic and secondary forms^1^. The incidence of pFSGS has been increasing especially in Western countries over the past years^2^. However, there are inconsistent data about the other countries from Europe, Asia, and Africa^3,4^. In addition, the immigrant Asian and Hispanic populations, resident in south-western of USA, have a higher frequency of pFSGS than the people who have been living in their country^5^. These data indicate that the pathogenesis of pFSGS may be influenced not only by age, race, and genetic but also environmental factors such as dietary habits, socio-cultural, socio-economic, and geographic features. In this manner, the determination of every nation’s characteristics may provide a valuable contribution for understanding the pFSGS pathogenesis and its accurate epidemiology. Turkish Society of Nephrology Glomerular Diseases Group (TSN-GOLD) published the prevalence of biopsy-proven primary glomerular diseases(PGDs) in 2014, in Turkey for the first time and, current data has also been published recently^6,7^. However, detailed information, dynamics of pFSGS, and trends have not been known deeply in Turkey yet. This study aimed to focus on the clinical, laboratory and histopathological characteristics of pFSGS and its trends in Turkish adults.

## Results

### Demographic, clinical and laboratory data

**Figure 1:**
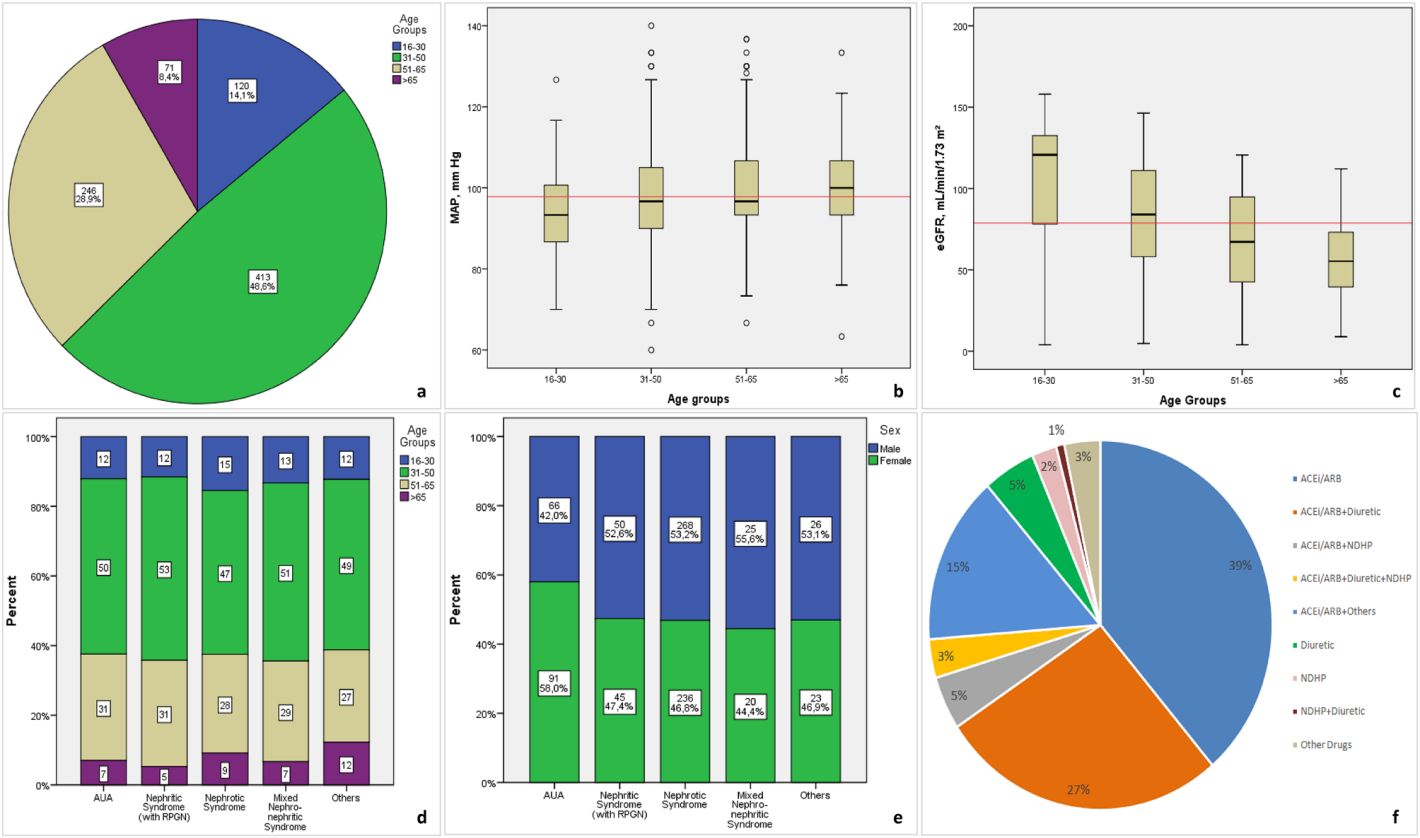
Age groups in all patients ((**a**); p > 0.10), means of the mean arterial pressure(MAP) ((**b**); p = 0.001, post hoc analysis showed 16–30 age-group was different from the other groups), eGFR ((**c**); p < 0.001, post hoc analysis showed 51–65 and > 65 age-group were similar statistically, while the other groups differed significantly from each other) in age groups, the distribution of age groups in biopsy indication groups ((**d**); p > 0.10), the gender in biopsy indication groups ((**e**); p < 0.05, Female sex was different in AUA group), and the frequencies of anti-hypertensive drug usage at the time of biopsy (**f**). (“*others”* means dihydropyridine group(DHP) of Ca^++^ channel blockers; *other drugs* means alpha receptor blockers-most of them- and the other antihypertensive dugs). Red lines show the mean of the parameters in all patients. The white chambers mean mild outliers, asterisks mean extreme outliers. *ACEi/ARB* angiotensin-converting enzyme inhibitors/angiotensin receptor blocker, *NDHP* non-dihydroprydine group of Ca^++^ channel blockers. The one-way ANOVA test with *Bonferroni *post hoc analysis was used to obtain p values in (**b**) and (**c**), Kruskal Wallis and Mann Whitney *U* tests were used to obtain the p values in (**d**) and (**e**). The frequencies of patients were shown used by pie graph and % in (**a**) and (**f**).

Of the 3875 patients enrolled in the TSN-GOLD PGDs database, 21.9% (850/3875) had pFSGS. The mean age of pFSGS patients at the time of kidney biopsy was 40.5 ± 14.2. Male gender was 51.2 (435/850) percent. 281 of patients (34.1%) had hypertension, 93 (11.5%) had diabetes, 64 (7.8%) had both. The mean value of BMI was 27.5 ± 5.5 kg/m^2^ for all patients. In total, 26.0% (115/442) had obesity (BMI ≥ 30 kg/m^2^). The laboratory findings of the patients on following; serum creatinine 1.0 mg/dL, (IQR = 0.7–1.4), albumin 3.4 ± 0.9 g/dL, and 24 h urinary protein 3400 mg/day, (IQR;1774–5740). In addition to proteinuria, the urinalysis revealed 32.6% of patients had hematuria, 15.2% had leukocyturia and 7.8% had both. The most common indication of kidney biopsy was nephrotic syndrome, accounting for 59.2% (504 of 850) in all patients, followed by AUA 18.5%, nephritic syndrome 11.2%, mixed nephrotic syndrome 5.3% (45/850) and others 5.8% (48/850) (Table 1). The majority of cases were in the age-period of 31–65 years (77.5%, n = 659) and 8.4% (n = 71) of patients were over 65 year-old (Fig. 1a). MAP and eGFR, which are known parameters related to age, were evaluated and found statistically different in age-groups(Fig. 1b,c). Biopsy indications were similar in all age groups(Fig. 1d). Female gender was more frequent in AUA group (Fig. 1e). The most frequently used antihypertensive drugs were angiotensin-converting enzyme inhibitors(ACEi) and/or angiotensin receptor blockers(ARB) with a usage rate of 88.6%, at the time of biopsy (Fig. 1f).

**Table 1:** Demographical, clinical and laboratory findings of all patients.

		n/n’	n	Patients
Demographic/clinical characteristics				
Age, year		850/0	850	40.5 ± 14.2
Gender		850/0		
Male			435	51.2
Female			425	48.8
Smoking		444/406		
Never smoked			230	51.8
Ex-smoker			90	20.3
Active smoker			124	27.9
Diabetes mellitus		850/0	93	11.5
Hypertension		817/33	281	34.4
DM and HT		817/33	64	7.8
Obesity		442/408	119	26.7
Pretibial edema		762/88	362	47.5
Biopsy indications		850/0		
Asymptomatic urine abnormalties			157	18.5
Nephritic syndrome			95	11.2
Nephrotic syndrome			504	59.2
Mixed Nephritic-Nephrotic			45	5.3
Others			49	5.8
BMI, kg/m^2^		442/418	442	27.5 ± 5.5
BMI categories	< 18.5		10	2.2
	18.5–24.9		141	31.9
	25–29.9		172	38.9
	30–34.9		72	16.3
	35–39.9		31	7.0
	> 39.9		12	2.7
SBP, mm Hg		747/103	747	130.8 ± 18.6
DBP, mm Hg		746/104	746	81.4 ± 11.4
MAP, mm Hg		746/104	746	106.0 ± 13.8
PP, mm Hg		746/104	746	49.3 ± 12.9
Hematuria		849/1	277	32.6
Leukocyturia		742/108	113	15.2
Hematuria and leucocyturia		741/109	58	7.8
Laboratory results				
Glucose, (mg/dL)		754/96	754	96,9 ± 32.9
BUN, (mg/dL)		808/42	808	21,1 ± 14.1
Creatinine, (mg/dL)		812/38	812	1.0 (0.7–1.4)
eGFR, (mL/min/1.73 m^2^)		812/38	812	82.1 ± 36.6
Albumin, (g/dL)		777/73	777	3.4 ± 0.9
Cholesterol, total (mg/dL)		647/203	647	264 ± 107.4
Triglyceride (mg/dL)		652/198	652	218.4 ± 135.2
HDL (mg/dL)		607/243	607	53.3 ± 22.3
LDL (mg/dL)		642/208	642	166.3 ± 87.3
Uric acid, (mg/dl)		707/143	707	6.3 ± 1.9
Hemoglobin (g/dL)		775/75	775	13,2 ± 2.0
ESR (mm/hour)		530/320	530	37.3 ± 28.0
Proteinuria (mg/day) (median, IQR)		759/101	759	3400 (1774–5740)
C3 complement, serum		584/266	584	31.3
Decreased			9	1.5
C4 complement, serum		573/277	573	32.6
Decreased			8	1.4

The data were presented as mean ± standard deviation, median (IQR, interquartile range), *n*’(missing data), *n*(%). *BMI* body mass index, *SBP* systolic blood pressure, *DBP* diastolic blood pressure, *MAP* mean arterial pressure, *PP* pulse pressure, *BUN* blood urea nitrogen, *eGFR* estimated glomerular filtration rate, *ESR* erythrocyte sedimentation rate.

### Histopathological data

The majority of renal biopsies were performed in nephrology clinics(68.9%), subsequently the interventional radiology clinics (27.2%), and unknown (outer center 3.9%) origin. The count of total glomeruli on biopsy were similar statistically (median, IQR; 14, 9–22; 14, 9–22 and 20, 11–28, respectively; p = 0.601). In histopathological examination, findings of LM, thickened basal membrane (TBM) (76.6%), mesangial proliferation (MP) (53.5%), global sclerosis (GS) (77.4%), tubular atrophy (TA) (67.5%), interstitial fibrosis (IF) (65.6%) and vascular changes(VC)(47.5%) were evaluated. The crescentic lesion described in 30 biopsy (4.5%). On IFM, IgG (7.2%), IgM (32.7%), IgA (9.1), C3 (32.9%) C1q (8.7%), fibrinogen(4.0%), κ(6.8%), and λ (7.3%) light chain staining positivity were included statistical analysis. The remaining parameter of C4 exclude from analysis because of the insufficient count of positivity. 23 (2.7%) of all patients had electron microscopic evaluation. The findings of LM and IFM were summarized in Table 2.

**Table 2:** Histopathological findings of kidney biopsies, light and immunfluorescence microscopy.

		n		Patients
Light microscopy				
Total glomeruli in biopsy samples		850		14 (9–22)
Mesengial proliferation, (MP)		796		426 (53.5)
Endocapillary proliferation		561		27 (4.8)
Exudative glomerular changes		550		41 (7.4)
Thickened basal membrane, (TBM)		792		607 (76.6)
Subendothelial immuncomplex deposition		686		8 (1.2)
Subepithelial immuncomplex deposition		682		16 (2.3)
Global sclerotic (GS)/total glomeruli		800		
Negative				181 (22.6)
< 25%				371 (46.4)
25–50%				183 (22.9
50–75%				55/(6.9)
> 75%				10 (1.2)
Segmental sclerotic (SS)/total glomeruli		850		
Undetermined				133 (15.6)
< 25%				581 (68.4)
25–50%				116 (13.6)
50–75%				17 (2.0)
> 75%				3 (0.4)
Crescentic		688		30 (4.5)
Cellular				11 (1.6)
Fibrocellular				10 (1.4)
Fibrous				9 (1.3)
Tubulary atrophy (TA)		799		
Negative				260 (32.5)
Grade 1 (< 25%)				405 (50.7)
Grade 2 (25–50%)				101 (12.7)
Grade 3 (> 50%)				33 (4.1)
Intersitial inflamation		850		519 (61.0)
Interstitial fibrosis (IF)		809		
Negative				278 (34.4)
Grade 1 (< 25%)				378 (46.7)
Grade 2 (25–50%)				118 (14.6)
Grade 3 (> 50%)				35 (4.3)
Vasculary changes (VC)		795		378 (47.5)
Electron microscopy		567		23 (2.7)
Immunofluorescence microscopy				
Ig G	+	789	48 (6.1)	
	+ +		9 (1.1)	
	+ + +		0 (0.0)	
	Negative		732 (92.8)	
Ig M	+	789	168 (21.3)	
	+ +		70 (8.9)	
	+ + +		20 (2.5)	
	Negative		531 (67.3)	
Ig A	+	788	58 (7.4)	
	+ +		13 (1.6)	
	+ + +		1 (0.1)	
	Negative		716 (90.9)	
C3	+	788	147 (18.6)	
	+ +		84 (10.7)	
	+ + +		28 (3.6)	
	Negative		529 (67.1)	
C1q	+	715	47 (6.6)	
	+ +		12 (1.7)	
	+ + +		3 (0.4)	
	Negative		653 (91.3)	
Kappa	+	463	27 (5.8)	
	+ +		3 (0.6)	
	+ + +		2 (0.4)	
	Negative		431 (93.1)	
Lambda	+	459	25 (5.4)	
	+ +		6 (1.3)	
	+ + +		3 (0.6)	
	Negative		425 (92.6)	
Fibrinogen	+	629	23 (3.5)	
	+ +		1 (0.2)	
	+ + +		2 (0.3)	
	Negative		603 (95.9)	

Data were presented as mean(± SD), median(IQR), and n(%) accordingly.

### Evaluation of the patients according to age

The patients were grouped as older than 40 year-old (427; 50.2%) and younger ones. Biopsy indications were similar in both groups.The frequency of active smokers was higher and comorbidities such as obesity, HT and DM were more common in older age group patients. Hemodynamic parameters like SBP, MAP, PP were also significantly higher than younger ones in those. On laboratory data, older age group patients had higher glucose, haemoglobin, and ESR; lower eGFR (75.7 ml/min/1.73m^2^). On LM, The MP and VC lesions were more seen and the rate of global sclerotic/total glomeruli in each biopsy speaceman was significantly higher in older patients. On IFM, C3 deposition trends to be higher in older patients (Table 3). This trend became statistically significant in over 65 year-old patients (35 positivity in 71 patients; p = 0.004).

**Table 3:** Comparison of the parameters between the patients below and over 40 years.

Parameters	n	< 40 years (n = 427)	n	≥ 40 years (n = 423)	p value
Demographic/clinical characteristics					
Smoking	226		218		0.005*
Never smoked		135 (0.60)		95 (0.44)	
Ex-smoker		34 (15.0)		56 (25.7)	
Active smoker		57 (25.2)		67 (30.7)	
Obesity	208	44 (21.2)	223	71 (31.8)	< 0.001*
Diabetes mellitus	427	16 (3.7)	423	77 (18.2)	< 0.001*
Hypertension	411	92 (22.4)	406	189 (44.6)	< 0.001*
SBP, mm Hg	371	127.8 (± 18.4)	375	133.7 (± 18.3)	** < 0.001**
MAP, mm Hg	371	96.5 (± 12.7)	375	99.1 (± 12.5)	**0.005**
PP, mm Hg	371	47.0 (± 12.9)	375	51.6 (± 12.6)	** < 0.001**
BMI, kg/m^2^	214	26.5 (± 5.6)	228	28.6 (± 5.6)	** < 0.001**
Laboratory analysis					
Glucose, (mg/dL)	383	92.0 (± 23.2)	371	102.1 (± 35.9)	** < 0.001**
BUN, (mg/dL)	410	18.5 (± 15.0)	398	23.8 (± 16.2)	** < 0.001**
Creatinine, (mg/dL)	365	0.9 (0.6–1.3)	408	1.1 (0.8–1.6)	0.020**
eGFR, (mL/min/1.73 m^2^)	365	82.4 (± 37.6)	408	75.7 (± 32.5)	** < 0.001**
Hemoglobin, (g/dL)	399	12.8 (± 2.1)	355	13.6 (± 1.9)	**0.009**
ESR (mm/hour)	256	32.3 (± 24.5))	274	42.0 (± 30.2)	** < 0.001**
Light microscopy findings					
Mesengial proliferation	398	198 (49.7)	398	228 (57.3)	0.033*
Vasculary changes	399	150 (37.6)	396	228 (57.6)	< 0.001*
Global sclerotic/total glomeruli	399		401		**0.003**^ð^
Negative		111 (27.8)		70 (17.5)	
< 25%		182 (45.6)		189 ( (47.1)	
25–50%		77 (19.3)		106 (26.4)	
50–75%		23 (5.8)		32 (8.0)	
> 75%		6 (1.5)		4 (1.0)	
Immunfluorescence microscopy findings					
C3 deposition	393	117 (29.8)	395	142 (35.9)	0.065*

Independent samples *t* test; *Chi-squared test; **Mann-Whithney *U* test; ^ð^Fisher’s exact test. Post hoc analysis were used if p < 0.05 in more than two groups. The data were presented as mean(± SD), median(IQR), and *n*(%). p < 0.05 is considered significant and presented in bold.

*SBP* systolic blood pressure, *MAP* mean arterial pressure, *PP* pulse pressure, *BMI* body mass index, *BUN* blood urea nitrogen, *eGFR* estimated glomerular filtration rate), *ESR* erythrocyte sedimentation rate.

### Evaluation of the patients according to gender

Almost one half of the patients (415 of the 850 patients, 48.8%) was female and had higher BMI (+ 1.6 kg/m^2^; p = 0.006), eGFR (+ 10.0 mL/min/1.73m^2^; p < 0.001) levels, lower active smoking rate, slightly lower SBP, DBP, MAP (− 2.2 mm Hg; p = 0.02). Females were more frequent in the AUA group (p = 0.046). The proteinuria was predominant (66.7%; 104/157) finding in patients with AUA indication. On laboratory serum creatinine, uric acid and 24-h urine protein were lower while eGFR, Hb, serum HDL, ALT and ESR levels were higher in females than males. On LM, there was no parameter that reached statistically significant but MP and the rate of segmental sclerotic/total glomeruli on biopsy speaceman had the trend to be higher than males. On IFM, IgM deposition was more common in females while IgG were in males (Table 4).

**Table 4:** Comparison of the parameters between males and females in study cohort.

Parameters	n	Male (n = 435)	n	Female (n = 415)	p value
Demographic/clinical characteristics					
Smoking	223		221		** < 0.001**^Δ^
Never smoked		76 (34.1)		154 (69.7)	
Ex-smoker		76 (34.1)		14 (6.3)	
Active smoker		71 (31.8)		53 (24.0)	
Hematuria	435	126 (29.0)	414	150 (36.2)	0.024*
Obesity	230	47 (20.4)	212	68 (32.1)	0.005*
SBP, mm Hg	381	132.2 (± 18.6)	366	129.3 (± 18.5)	**0.032**
DBP, mm Hg	380	82.3 (± 11.9)	366	80.5 (± 10.7)	**0.026**
MAP, mm Hg	380	98.9 (± 12.9)	366	96.7 (± 12.3)	**0.022**
BMI, kg/m^2^	230	26.8 (± 4.7)	212	28.4 (± 6.6)	**0.006**
Laboratory analysis					
BUN, (mg/dL)	408	23.5.6 (± 15.6)	394	18.6 (± 14.0)	** < 0.001**
Creatinine, (mg/dL)	412	1.2 (0.9–1.7)	400	0.8 (0.6–1.2)	< 0.001**
eGFR, (mL/min/1.73 m^2^)	412	73.9 (± 34.0)	400	83.9 (± 35.4)	** < 0.001**
Uric acid, mg/dL	361	6.8 (± 1.8)	346	5.8 (± 1.8)	** < 0.001**
HDL, mg/dL	309	47.8 (± 17.6)	298	59.0 (± 25.0)	** < 0.001**
ALT, IU/L	368	22.8 (± 17.6)	354	19.2 (± 11.8)	**0.002**
Hemoglobin, (g/dL)	388	14.0 (± 2.0)	387	12.4 (± 1.7)	** < 0.001**
ESR (mm/hour)	277	33.0 (± 28.5)	253	41.8 (± 26.9)	** < 0.001**
Proteinuria, mg/day	383	3570 (1918–6975)	374	3200 (1627–5226)	**0.002**
Light microscopy findings					
Mesengial proliferation	409	206 (49.6)	387	220 (56.8)	0.067*
Segmental sclerotic/total glomeruli	407		386		0.084^ð^
Undetermined		27 (6.6)		30 (7.7)	
< 25%		320 (78.6)		323 (83.7)	
25–50%		71 (17.4)		58 (15.0)	
50–75%		14 (3.4)		4 (1.0)	
> 75%		2 (0.5)		1 (0.3)	
Immunfluorescence microscopy findings					
IgM	409	120 (29.3)	380	138 (36.3)	0.037*
IgG	410	37 (9.0)	379	20 (5.3)	0.042*

*SBP* systolic blood pressure, *DBP* diastolic blood pressure, *MAP* mean arterial pressure, *PP* pulse pressure, *BMI* body mass index, *BUN* blood urea nitrogen, *eGFR* estimated glomerular filtration rate, *ESR* erythrocyte sedimentation rate.

Independent samples *t* test *Chi-squared test; **Mann-Whithney *U* test, ^ð^Fisher’s exact test. The data were presented as mean(± SD), median(IQR), n(%).

Significant values are in bold.

### Evaluation of the patients according to BMI

In total, 442 patients had BMI data and the analysis was performed in those 115 of 442 (26%) patients had obesity and they were older than non-obese patients (43.6 years to 39.5 years). HT and DM were more common and SBP, MAP, PP also significantly higher in obese patients. There was no difference in terms of biopsy indications in those. On laboratory data, obese patients had higher glucose, ALT levels. On LM, there was a trend to be higher frequency of TBM, TA and to be higher rate of global sclerotic glomeruli. On IFM, C3 deposition was more frequent finding in obese patients (Table 5).

**Table 5:** Comparison of the parameters between the patients with and without obesity (according to BMI).

Parameters	n	BMI < 30 kg/m^2^ (n = 327)	n	BMI ≥ 30 kg/m^2^ (n = 115)	p value
Demographic/clinical characteristics					
Age, year	327	39.5 (± 14.6)	115	43.6 (± 11.5)	**0.003**
Gender	327		115		0.001*
Male		183 (56.0)		47 (40.9)	
Female		144 (44.0)		68 (59.1)	
Diabetes mellitus	327	30 (9.2)	115	24 (20.1)	0.001*
Hypertension	324	78 (24.1)	115	42 (36.5)	0.010*
Pretibial edema	324	160 (49.4)	115	68 (59.1)	0.072*
SBP, mm Hg	324	130.2 (± 17.8)	115	136.4 (± 17.0)	**0.001**
DBP, mm Hg	324	81.0 (± 11.7)	115	83.4 (± 10.4)	0.059
MAP, mm Hg	324	97.4 (± 12.9)	115	101.0 (± 11.5)	**0.008**
PP, mm Hg	324	49.2 (± 12.0)	115	53.0 (± 13.1)	**0.004**
BMI, kg/m^2^	327	25.0 (± 3.2)	115	34.8 (± 5.0)	** < 0.001**
Laboratory analysis					
Glucose, (mg/dL)	319	95.5 (± 31.2)	111	105.7 (± 35.0)	**0.005**
Creatinine, (mg/dL)	325	1.0 (0.7–1.2)	114	0.9 (0.7–1.2)	0.055**
Uric acid, mg/dL	307	6.2 (± 1.9)	104	6.6 (± 1.8)	0.052
Albumin, (g/dL)	321	3.3 (± 1.0)	111	3.5 (± 0.8)	0.089
ALT, IU/L	308	19.3 (± 11.4)	106	24.1 (± 16.5)	**0.006**
Light microscopy findings					
Thickened basal membrane	319	75 (23.5)	110	15 (13.6)	0.070*
Global sclerotic/total glomeruli	317		112		0.058^ð^
Negative		76 (24.0)		17 (15.2)	
< 25%		139 (43.8)		65 (58.0)	
25–50%		71 (22.5)		23 (20.5)	
50–75%		28 (8.8)		5 (4.5)	
> 75%		3 (0.9)		2 (1.8)	
Tubulary atrophy	317		111		0.068^ð^
Negative		105 (33.1)		42 (37.8)	
Grade 1 (< 25%)		153 (48.3)		59 (53.2)	
Grade 2 (25–50%)		48 (15.1)		6 (5.4)	
Grade 3 (> 50%)		11(3.5)		4 (3.6)	
Immunfluorescence microscopy findings					
C3	297	107 (28.5)	41	18 (43.9)	0.042*

*SBP* systolic blood pressure, *DBP* diastolic blood pressure, *MAP* mean arterial pressure, *PP* pulse pressure, *BMI* body mass index.

Independent samples *t* test *Chi-squared test; **Mann-Whithney *U* test; ^ð^Fisher’s exact test. The data were presented as mean(± SD), median(IQR), and *n*(%).

Significant values are in bold.

### Evaluation of the patients according to presence of HT

In total, 817 patients had blood pressure measurement correctly and 34.4% (n = 281) of those had HT, 23.1% (n = 65) were diabetic. Hypertensive patients had older age, higher BMI, serum glucose, creatinine, uric acid, albumin levels and lower eGFR, serum total cholesterol, LDL, HDL levels. On LM, VC, TA, IF were more frequent lesions. The frequency of global sclerotic glomerulus was significantly higher in hipertansive patients. On IFM, there was no significant difference statistically (Table 6).

**Table 6:** Comparison of the parameters between the patients with or without HT.

Parameters	n	HT ( +), (n = 281)	n	HT (–), (n = 536)	p value
Demographic/clinical characteristics					
Age, year	281	46.0 (± 13.6)	536	37.4 (± 13.5)	** < 0.001**
Diabetes mellitus	281	65 (23.1)	536	26 (4.9)	< 0.001*
Biopsy indications	281		536		**0.019**^Δ^
Asymptomatic urine abnormalties	151	68 (45.0)	151	83 (55.0)	
Nephritic syndrome	92	31 (33.7)	92	61 (66.3)	
Nephrotic syndrome	486	148 (30.5)	486	338 (69.5)	
Mixed nephritic-nephrotic	41	15 (36.6)	41	26 (63.4)	
Others	47	19 (40.4)	47	28 (59.6)	
SBP, mm Hg	244	137.0 (± 19.7)	479	127.5 (± 17.2)	** < 0.001**
DBP, mm Hg	243	84.3 (± 10.9)	479	79.8 (± 11.3)	** < 0.001**
BMI, kg/m^2^	146	28.4 (± 5.1)	288	27.1 (± 5.9)	**0.020**
Laboratory analysis					
Glucose, (mg/dL)	245	102.8 (± 38.2)	488	94.5 (± 26.0)	**0.002**
BUN, (mg/dL)	267	48.8 (± 29.4)	512	41.2 (± 31.5)	**0.001**
Creatinine, (mg/dL)	267	1.2 (0.8–1.6)	516	0.9 (0.7–1.3)	< 0.001**
eGFR, (mL/min/1.73 m^2^)	267	66.5 (± 31.3)	516	85.3 (± 35.4)	** < 0.001**
Uric acid, mg/dL	235	6.7 (± 1.8)	451	6.1 (± 1.8)	** < 0.001**
Cholesterol, total (mg/dL)	215	245.1 (± 83.2)	413	305.8 (± 116.6)	** < 0.001**
HDL, mg/dL	205	49.0 (± 16.4)	385	55.8 (± 24.8)	** < 0.001**
LDL (mg/dL)	213	152.0 (± 66.2)	410	173.0 (± 94.7)	**0.001**
Albumin, (g/dL)	239	3.6 (± 0.8)	496	3.3 (± 1.0)	** < 0.001**
Hemoglobin, (g/dL)	255	13.0 (± 2.0)	387	13.3 (± 2.0)	0.097
Light microscopy findings					
Vasculary changes	266	164 (61.7)	503	228 (40.4)	< 0.001*
Thickened basal membrane	281	74 (26.3)	536	102 (19.0)	0.051*
Global sclerotic/total glomeruli	267		510		** < 0.001**^ð^
Negative		32 (12.0)		145 (28.4)	
< 25%		118 (44.2)		246 (48.2)	
25–50%		83 (31.1)		90 (17.6)	
50–75%		28 (10.5)		25 (4.9)	
> 75%		6 (2.2)		4 (0.8)	
Tubulary atrophy	267		507		** < 0.001**^Δ^
Negative		67 (25.1)		189 (37.3)	
Grade 1 (< 25%)		136 (50.9)		253 (49.9)	
Grade 2 (25–50%)		45 (16.9)		52 (10.3)	
Grade 3 (> 50%)		19 (7.1)		13 (2.6)	
Interstitial fibrosis	265		504		** < 0.001**^Δ^
Negative		75 (28.3)		180 (35.7)	
Grade 1 (< 25%)		113 (42.6)		254 (50.4)	
Grade 2 (25–50%)		57 (21.5)		56 (11.1)	
Grade 3 (> 50%)		20 (7.5)		14 (2.8)	
Immunfluorescence microscopy findings					
IgM	189	77 (28.9)	327	170 (34.2)	0.081*
C1q	233	17 (6.8)	402	45 (10.1)	0.093*

*SBP* systolic blood pressure, *DBP* diastolic blood pressure, *BMI* body mass index, *BUN* blood urea nitrogen, *eGFR* estimated glomerular filtration rate.

Independent samples *t* test; *Chi-squared test; **Mann-Whithney *U* test; ^ð^Fisher’s exact test; Kruskal Wallis test^Δ^. The data were presented as mean(± SD), median(IQR), and *n*(%). Post hoc analysis were used if p < 0.05) in more than two groups.

Significant values are in bold.

### Evaluation of the patients according to eGFR

The patients were grouped according to eGFR as above and below 45 mL/min/1.73 m^2^. Lower eGFR group (46.3 years) was older than higher one (38.8 years) with male gender predominancy (60.2%). SBP and PP were significantly higher in this group. On laboratory, BUN, creatinine, uric acid levels were higher and hb levels were lower as expected. On LM, TA, VC and global sclerotic glomeruli were more seen. On IFM IgM deposition was more common in higher eGFR group than the lower one (Table 7).

**Table 7:** Comparison of the parameters between the patients with eGFR < 45 and eGFR ≥ 45 mL/min/1.73 m^2^.

Parameters	n	eGFR < 45 (n = 166)	n	eGFR ≥ 45 (n = 646)	p value
Demographic/clinical characteristics					
Age, year	166	46.3 (± 14.4)	646	38.8 (± 13.7)	** < 0.001**
Gender	812		812		0.006*
Male	412	100 (60.2)	412	312 (48.3)	
Female	400	66(39.8)	400	334 (51.7)	
SBP, mm Hg	154	134.5 (± 19.1)	588	129.9 (± 18.3)	**0.006**
MAP, mm Hg	153	99.6 (± 12.9)	588	96.8 (± 12.3)	0.064
PP, mm Hg	153	51.6 (± 13.0)	588	48.7 (± 12.9)	**0.015**
Laboratory analysis					
BUN, (mg/dL)	164	38.2 (± 21.6)	636	16.7 (± 8.3)	** < 0.001**
Creatinine, (mg/dL)	166	2.1 (1.8–3.0)	646	0.9 (0.7–1.2)	< 0.001**
eGFR, (mL/min/1.73 m^2^)	166	29.9 (± 11.3)	646	91.4 (± 27.2)	** < 0.001**
Uric acid, mg/dL	141	7.4 (± 2.0)	565	6.0 (± 1.7)	** < 0.001**
HDL, mg/dL	129	50.0 (± 26.0)	476	54.2 (± 21.1)	0.096
ALT, IU/L	147	18.4 (± 12.7)	573	21.8 (± 15.6)	**0.015**
Hemoglobin, (g/dL)	157	12.2 (± 2.0)	615	13.5 (± 1.9)	** < 0.001**
ESR (mm/hour)	114	45.0 (± 32.4)	415	35.1 (± 26.2)	**0.003**
Light microscopy findings					
Tubular atrophy	156		610		< 0.001*
Negative		25 (16.0)		222 (36.4)	
< 25%		67 (42.9)		322 (52.8)	
25–50%		45 (28.8)		53 (8.7)	
> 50%		19 (12.2)		13 (3.3)	
Vasculary Changes	159	51 (42.1)	604	350 (57.9)	< 0.001*
Segmental sclerotic/total glomeruli	160		606		0.093^ð^
Negative		0 (00.0)		0 (00.0)	
< 25%		123 (76.69)		502 (82.8)	
25–50%		30 (18.8)		92 (15.2)	
50–75%		5 (3.1)		11 (1.8)	
> 75%		2 (1.3)		1 (0.2)	
Global sclerotic/total glomeruli	158		612		** < 0.001**^ð^
Negative		21 (13.3)		151 (24.7)	
< 25%		51 (32.3)		310 (50.7)	
25–50%		55 (34.8)		119 (19.4)	
50–75%		24 (15.3)		29 (4.7)	
> 75%		7 4.4)		3 (0.5)	
Immunfluorescence microscopy findings					
IgM	150	35 (23.3)	611	213 (34.9)	0.007*

*SBP* systolic blood pressure, *DBP* diastolic blood pressure, *BUN* blood urea nitrogen, *eGFR* estimated glomerular filtration rate, *ESR* erythrocyte sedimentation rate.

Independent samples t-test; *Chi-squared test; **Mann-Whithney *U* test; ^ð^Fisher’s exact test. The data were presented as mean(± SD), median(IQR), and *n*(%).

Significant values are in bold.

### Evaluation of the patients according to proteinuria

Pretibial edema was more frequent in nephrotic proteinuric patients while hematuria was in nephritic proteinuric ones as expected. Nearly one third of patients was hematuric in nephrotic proteinuric group. Serum albumin was lower while lipids, and ESR were significantly higher in those. Hemodynamic parameters and eGFR with serum creatinine were similar in both groups. On LM, TBM, the frequency of global sclerotic glomerulus were significantly higher in nephritic proteinuric patients. On IFM, kappa cummulation were frequent in nephrotic proteinuric group. There was also a trend to be higher of C1q depositon (Table 8).

**Table 8:** Comparison of the parameters between the patients with nephritic and nephrotic proteinuria.

Parameters	n	< 3.5gr/day, (n = 396)	n	≥ 3.5 gr/day, (n = 363)	p value
Demographic/clinical characteristics					
Pretibial edema	364	109 (29.9)	351	229 (65.2)	< 0.001*
Hematuria	396	152 (38.4)	362	105 (29.0)	0.006*
Laboratory analysis					
Albumin, (g/dL)	375	3.8 (± 0.7)	353	2.9 (± 0.9)	** < 0.001**
Cholesterol, total (mg/dL)	310	225.3 (± 65.9)	297	305.8 (± 122.9)	** < 0.001**
Triglyceride (mg/dL)	304	193.3 (± 112.8)	307	249.0 (± 147.2)	** < 0.001**
HDL, mg/dL	283	50.1 (± 19.8)	288	56.1 (± 24.5)	**0.001**
LDL (mg/dL)	305	135.3 (± 48.1)	299	197 (± 104.0)	** < 0.001**
ESR (mm/hour)	250	29.3 (± 24.1)	256	44.1 (± 29.1)	** < 0.001**
Light microscopy findings					
Thickened basal membrane	396	70 (17.7)	363	89 (24.5)	0.007*
Segmental sclerotic/total glomeruli	429		421		0.051^ð^
Undetermined		67 (15.6)		66 (15.7)	
< 25%		310 (72.3)		277 (65.8)	
25–50%		43 (10.0)		68 (16.1)	
50–75%		8 (1.9)		8 (1.9)	
> 75%		1 (0.2)		2 (0.5)	
Global sclerotic/total glomeruli	368		352		** < 0.001**^ð^
Negative		65 (17.7)		95 (27.0)	
< 25%		185 (50.3)		153 (43.5)	
25–50%		89 (24.3)		74 (21.0)	
50–75%		24 (6.5)		26 (7.4)	
> 75%		5 (1.4)		4 (1.1)	
Immunfluorescence microscopy findings					
C1q	335	23 (6.9)	320	34 (10.6)	0.088*
Kappa	230	10 (4.3)	233	22 (9.4)	0.031*

*ESR* erythrocyte sedimentation rate.

Independent samples *t* test; *Chi-squared test; ^ð^Fisher’s exact test. The data were presented as mean(± SD) and *n*(%).

Significant values are in bold.

### Correlations of histopathologic findings with clinicodemographic data

In LM findings both TA and IF were more frequent among men. The patients with VC and/or MP have older age than negative controls. All parameters regarding arterial blood pressure were significantly higher in GS and VC positive patients. In laboratory analysis, serum creatinine levels were significantly different and correlated positively with positivities of TBM, GS, TA, IF, and VC. Serum uric acid(sUA) levels were higher in the patients with GS, TA, IF, and VC and positively correlated with the severity of the lesions. Serum hemoglobin levels trend to be lower in patients with TA and negatively weak correlated with its severity. Erythrocyte sedimentation rate(ESR) was higher in patients with SS and positively correlated with its frequency. Proteinuria was higher in the patients who have TBM, GS, SS and was correlated with the percentage of involved glomeruli (Table 1Supp). Hematuria was observed more frequent in patients with at least one positive staining of IgG, IgM, IgA, and C1q in their biopsy specimens. Leukocyturia was higher only in patients with IgA positivity. IgM positivity was correlated with lower eGFR, serum albumin, and higher serum total cholesterol, LDL, UA, and proteinuria (Table 2Supp).

### Trends of pFSGS patients’ characteristics

The FSGS patients in the 2012–2019 period had older age, less pyuria, more bodyweight, higher serum glucose, and albumin levels, lower HDL and ESR levels when compared with the patients in 1994–2012. On LM, MP and TBM were more frequent in former group while the frequency and severity of interstitial fibrosis was significantly higher in latter. On IFM, IgG and C3 depositions were more common in former group (Table 9).

**Table 9:** The comparison of 1994–2019 and 2012–2019 cohorts.

Parameters	n	1994–2012, data n = 481	n	2012–2019, data n = 615	P
Demographics n (%)					
Age, year	481	36.1 (± 13.3)	615	42.1 (± 14.1)	** < 0.001**
Leukocyturia	167	35 (21.0)	575	78 (13.6)	0.019*
Clinical characteristics (mean ± SD)					
Weight, kg	100	73.9 (± 17.1)	342	78,2 (± 16.1)	**0.014**
BMI, kg/m^2^	100	26.6 (± 5.6)	342	27.8 (± 5.5)	0.059
Hypertension	203	56 (27.6)	614	255 (36.6)	0.018*
Diabetes mellitus	210	5 (2.4)	614	88 (13.5)	< 0.001*
Laboratory analysis (mean ± SD)					
Glucose, (mg/dL)	174	90.3 (± 19.0)	580	99.0 (± 33.0)	** < 0.001**
Albumin, (g/dL)	385	3.3 (± 0.9)	592	3.4 (± 0.9)	**0.049**
Triglyceride (mg/dL)	156	203.3 (± 126.7)	496	223 (± 137.5)	0.096
HDL (mg/dL)	138	56.6 (± 22.1)	469	52.3 (± 22.2)	**0.047**
ESR (mm/hour)	117	43.2 (± 33.2)	413	37 (± 26.1)	**0.026**
Biopsy indications	232		608		0.073^Δ^
Asymptomatic urine abnormalties		33 (14.2)		124 (20.4)	
Nephritic syndrome		31 (13.4)		64 (10.5)	
Nephrotic syndrome		143 (61.6)		361 (59.4)	
Mixed nephritic-nephrotic		18 (7.8)		27 (4.4)	
Others		7 (3.0)		32 (5.3)	
Light microscopy findings					
Vasculary changes	399	150 (37.6)	396	228 (57.6)	0.097*
Mesengial proliferation	210	134 (63.8)	586	292 (49.8)	< 0.001*
Thickened basal membrane	207	56 (27.0)	585	129 (22.0)	0.001*
Interstitial fibrosis	213		582		**0.003**^**Δ**^
Negative		87 (40.8)		177 (30.4)	
< 25%		87 (40.8)		291 (50.0)	
25–50%		27 (12.7)		91 (15.6)	
> 50%		12 (5.6)		23 (4.0)	
Immunfluorescence microscopy findings					
IgG deposition	416	180 (43.4)	585	170 (29.0)	< 0.001*
C3 deposition	420	183 (43.6)	577	204 (35.4)	0.030*

*BMI* body mass index, *BUN* blood urea nitrogen.

Independent *t*-test. *Chi-square test, Kruskal Wallis test^Δ^. Post hoc analysis were used if p < 0.05) in more than two groups.

Significant values are in bold.

## Discussion

Here we report that the prevalence of pFSGS is 21.9% with the relatively similar sex rates (M/F; 51.2%/48.8%) and nephrotic syndrome (59.5%) is the most frequent clinical presentation of pFSGS in Turkey. The epidemiology of pFSGS has interesting survey in the different part of the world in last two decades. Some studies reported that the incidence of pFSGS has been increasing in particularly USA^3,4,8^. Its prevelance is almost 15–30% in adults between 15 and 60 years of age, and increases to 30–35% over 60 years of age. When diabetic glomerular diseases are excluded, pFSGS was found to be the most common cause of ESRD in both white and black^4,8^. However, there are inconsistent data about the other countries from Europe (14.9%), Asia (6.9%), and Latin America (15.8%)^9^. Similarly, in China, the prevalence of pFSGS has shown a decreasing trend in the last decade, according to a recent study^10^. This higher prevelance and slight increase from 19.3 to 21.9% indicate that Turkish adults have different characteristics from the Asian population in terms of pFSGS dynamics.

The frequency of hematuria, leukocyturia and both are 32.6%, 15.2%,and 7.8%, respectively. These findings are consistent with the majority of the FSGS studies. It has been known for four decades that nephrotic presentation of pFSGS is common in adults, and more common in children^11^. Additionally, hematuria is frequently (approximately 40%) associated with the finding in adulthood nephrotic syndrome. Although the clinical significance of leukocyturia is unclear, hematuria in FSGS may be associated with poor renal outcome^12^. Some studies implicated that hematuria regarding FSGS may be related to GBM abnormality and/or MP on the biopsy^13,14^. In the present study, MP and TBM are not related to hematuria. But it is more frequent in patients with GS lesions. Similarly, immunologic depositions in the kidney such as Ig G, M, A, and C1q positivity on IFM are associated with hematuria.

Sex and age seem to be important parameters in FSGS prognosis according to the literature. Many studies emphasized that male FSGS patients have a worse outcome than females although some studies have inconsistent conclusions. The Canadian study consistedthe follow-up of 370 patients with FSGS revealed a worse clinical course in men than women, even in as shorter as 12 months follow-up period, in Toronto Glomerulonephritis registry^15^. Both gender groups were similar statistically in terms of BMI, MAP and age at baseline. Conversely, another study, had 15 years follow-up period, showed that gender, age, and BMI have no effect on these parameters^16^. A recent study from Japan showed that age is positively correlated with SBP, BMI; negatively with eGFR^17^. Although the design of the present study has no follow-up period, females have higher BMI, eGFR and lower MAP statistically at biopsy period. Age is also correlated with these clinical parameters (positively with BMI, SBP, MAP, PP and negatively with eGFR) consistent to Canadian and Japanese studies.

The mean BMI is 27.5 ± 5.5 kg/m^2^ in the present cohort and the majority of cases are in the age group of 31–65 years (77%). The relationship between obesity and FSGS was first described in 1974 and this fenomenon has been considered as a secondary cause of FSGS for several decades^18^. In the present study, biopsy specimens are carefully evaluated with clinical data by the pathologist and nephrologist to distinguish primer forms from the secondary ones. Supportively, when the present and the TURDEP-2 study, conducted in the general population aged over 18 years in Turkey in 2010, compared interms of the obesity rates and BMI data, it is observed that pFSGS patients in the present study have lower results (31.9%, 28.6 kg/m^2^; 26.0%, 27.5 kg/m^2^, respectively) than the normal population. The rates of class II (35–40 kg/m^2^) and class III(> 40 kg/m^2^) obesity were also lower in pFSGS patients than in thoseTURDEP-2 cohort (7.0%, 8.8%; 2.7%, 7.2% respectively)^19^.

Hypertension (HT) is one of the important factor alone for a worse prognosis and rapid progression of kidney disease in all glomerulonephritis and also pFSGS. In the present cohort, approximately one-third (34.4%) of patients is hypertensive. This rate is higher than the prevelance of HT (30.3%) found in PATENT-2 study conducted in theTurkish adult population^20^. However, it is lower than in many FSGS studies in the literature. The study in the Japanese population showed that the prevelance of HT was 56.2% in pFSGS patients(n = 996). However, the mean age was 58 years in those and significantly higher than present cohort (40.5 years)^17^. In furtherance, the HT prevelance was increased to 45.9% in 50–65 year-old group and to 60.3% in older than 65 year-old group in the present study. The age looks like the major contributor for developing HT in the both study populations. Additionally, there are significantly decreased eGFR, increased serum creatinine, glucose, uric acid and DM frequency in hypertensive patients in present cohort. On LM, chronic lesions like VC, TBM, TA, and IF were also more seen. Beside the HT, cumulation of diseases with aging and increasing metabolic load may be cause of these findings.

The patients in decreased eGFR (< 45 mL/min/1.73 m^2^) group had high levels of SBP and PP compared the  ≥ 45 mL/min/1.73 m^2^ ones. They had also older age, higher serum uric acid, ESR and lower haemoglobin levels. On LM, TA, VC and GS were the more frequent findings. Global sclerosis and VC were positively correlated with the components of arterial blood pressure in this group. The study from China showed that tubule-interstitial and vascular lesions are more common pathologic lesions in the patients with higher glomerulosclerosis score and pFSGS is on the third rank after DM and IgA nephropathy in terms of the frequency of tubulointerstitial lesions. The authors concluded that the clinical significance of these lesions in those patients should be elucidated in the progression of CKD^21^. Some studies support this conclusion with the hypothesis of a decreased area of the effective capillary network due to arteriolar lesions that is correlated with tubule-interstitial lesions and interstitial fibrosis with the possible hypoxia mechanism^22,23^. Indeed, a more recent clinical study revealed that IF, TA, and interstitial inflammation at diagnosis were related to worse long-term renal survival in FSGS patients^16^. These data implicate that not only glomerular but also tubulointerstitial-vascular lesions might have significance clinically in FSGS. Consistent with the China study mentioned above, TA and IF were more frequent with the higher severity index in male gender in the present study. Over the last decade, the need for new strategies beyond older classifications based solely on glomerular lesions has been expressed in the growing body of evidence. Recent study support this conclusion in the patients with minimal change disease and FSGS^24^.

Typical lesion of pFSGS is SS and typical clinical presentation is abrupt edema, hypoalbunemia and nephrotic proteinuria according to literature. Interestingly, in the present study, only SS, specific lesion of pFSGS, had a correlation with ESR. High ESR level is a nonspecific finding and can be seen not only in variety of disease condition but also in healthy females and elderly adults. However some of findings in the present study implicate that there may be a different clinical meaning of ESR in pFSGS. For instance, nephrotic proteinuric group which was similar with nephritic one according to age, gender, and eGFR also had higher ESR in the present study. The summation effects that belongs to different components like coagulation factors, alfa-2 macroglobulin, plasminogen, and especially fibrinogen in plasma may cause this entity^25^. Unlikely, sUA level is only correlated with GS which is a nonspecific lesion of glomerular diseases. It is well known that the sUA level could be affected both by decreased kidney function and by many factors such as genetic, metabolic, and environmental manner. According to some studies sUA is also a risk factor for the development of CKD via HT, DM, obesity, etc. that also causes of secondary FSGS^26^. In this study, sUA was positively correlated with serum creatinine levels, thus it could be unabled to conclude whether a cause or consequence for CKD.

In IFM findings, IgM positivity is positively correlated with lower eGFR, serum albumin and higher serum total cholesterol, LDL,and proteinuria. In the adriamycin-induced FSGS animal model, it is shown that IgM could activate the complement cascade and contribute to the CKD progression in FSGS^27,28^. Recently, Zhang et al*.* have shown that IgM accumulation in kidney is associated with a worse prognosis in Chinese pFSGS patients, especially with the presence of accompanying C3 deposits^29^. Similarly, Miroğlu et al. revealed the worse outcome of the pFSGS patients who had co-depositon of IgM and C3 on their biopsy speaceman in Turkey^30^. Findings of the present study were consistent with these studies in terms of IgM depositions at first admission. In our cohort the IgM positivity was 32.7% and lower from both aformantioned Chinese (54.7%) and Turkish (51.1%) studies. More recently the frequency of Ig M deposition was reported lower in Pakistanip FSGS patients (15.5%)^31^. The mean ages of depositions positive patients in aforementioned studies were also different (26 in Chinese, 36 in Turkish, ~ 30 in Pakistani and 42 years in the present cohort). These findings implicate that ethnicity and age may affect the IFM findings. Considering the study by Miroğlu et al. the presence of significant differences in IFM findings even two samples from Turkey demonstrates the socio-cultural-economic status, enviremental and other local factors may be a modifier to accumulate of immune markers in the kidney in pFSGS patients. A study also showed that low serum C3 levels are associated with C3 deposition in the kidney and C3 deposition alone leads worse renal survival in pFSGS patients^32^. However, the presence of C3 deposition alone is not related to any laboratory or histopathological results in our study. Interestingly, there is only a weak positive correlation of C3 deposition with DBP and MAP. There is no data in the literature, according to our knowledge, about the relationship between C3 deposition and arterial blood pressure in pFSGS. But in a study, it is found that C3 deposits were correlated with SBP in IgA nephropathy, though the mechanism is unclear^33^.

We found that the metabolic markers such as serum fastig glucose levels and BMI had been increasing when compared 1994–2012 and 2012–2019 gorups in the present study. Supportingly, according to the results of TURDEP study mentioned above, the metabolic parameters (DM, obesity, impaired glucose tolearance rate) are getting worse through 1997 to 2010 in Turkish adult population^19^. In addition, the 2012–2019 cohort has higher HT rate, and older age than the former. This observation brings to mind the likelihood of the misdiagnosis of secondary FSGS cases in the study cohort. However, most studies are retrospective like present one and have higher heterogeneity in terms of including criteria, biopsy policies, methodologies, population characteristics such as age, race, ethnicity, socio-economic conditions, etc. In light of these data, we thought that the high prevalence of pFSGS in some studies might be due to a limited analysis of the secondary forms.

Despite having a large population, its retrospective and cross-sectional nature were the limitations of this study. In addition, only a limited number of biopsies is evaluated with EM and the pFSGS diagnosis were confirmed only by a local pathologist alone. The Colombia classification could not be used to evaluate the biopsies, although detailed histopathological data were presented. This may be a consequence of the different approaches of pathologists of the centers participating in this study and the absence of acceptable standardization for evaluating glomerulonephritis across our country yet. However, as mentioned above, in this period when the importance of extraglomerular histopathological findings of pFSGS is better understood, the value of such data will increase for novel classifications.

Briefly, this study shows the status of pFSGS in terms of epidemiologic, clinicodemographic and histopathological features of adult patients in Turkey. The prevalence of pFSGS in Turkish adults over the past decade seems in steady state with a slight increase. Although there are some differencies, the characteristics of pFSGS patients in Turkey, show parallelism with those seen in Western countries.

## Materials and methods

It is a retrospective, multicenter, cross-sectional cohort study. The data were obtained from the National Primary Glomerular Diseases registry system. The web-based software of the registry system was constituted by the Turkish Society of Nephrology for surveillance and improving the outcome of PGDs in Turkey. The registration process has been going on since 1994 and today 47 nephrology centers from seven geographical regions of Turkey have joined to the registry system. During this pocess, the data at the time of admission resulted with kidney biopsy, were recorded according to the relevant investigator’s declaration. Since each patient was from different centers, hematologic/biochemical(glucose, BUN, creatinine, albumin, triglyceride, HDL/LDL cholesterol, uric acid, calcium, hemoglobin, erythrocyte sedimentation rate, proteinuria), immunological (ANA, ANCA, C3, C4) and serological(hepatitis B and C and HIV Ab) analysis were studied in the different laboratories. 4399 of PGDs records were scanned between May 2009 and June 2019 and 524 of those were excluded because of insufficient data. Of the remaining 3875 PGDs records, patients whose kidney biopsies were reported as FSGS by the nephropathologist were evaluated in-depth by the study team. Having the one of following features such as positive genetic test results with family history of kidney disease, determined viral infection like HIV, parvo virus, etc., medication with drugs that could be related the FSGS and prior kidney disease (obesity-related kidney disease, reflux nephropathy, an operation or a disease that could be cause of renal mass reduction etc.) before FSGS diagnosis considered as exclusion criteria. If a diabetic patient had typical diabetic-related lesion like nodular or diffuse mesangial sclerosis with proliferative retinopathic findings and a hypertensive patient had typical findings of grade 3 (hemorrhagic and/or exudative) 4 (pupil eudema) retinopathy were also excluded. Following these exclusions, 850 patients (2009–2012) were considered to have pFSGS and were taken the further evaluation. The data of pFSGS patients obtained in 1994–20,012 (n = 481) and 2012–2019 (n = 615) were also compared to determine the trends,.

### Data collection and definitions

The demographic and clinical data of 850 patients were obtained from database. The presence of hematuria was described as erythrocyte ≥ 5/HPF and leukocyturia was diagnosed as leukocyte ≥ 5/HPF in the urine sediment. Indications of kidney biopsy were categorized clinically as follows; nephrotic syndrome, nephritic syndrome including rapidly progressive glomerulonephritis(RPGN), mixed nephrotic syndrome, asymptomatic urinary abnormalities(AUA), and others^6,7^. Nephrotic syndrome was defined by the presence of proteinuria (protein excretion greater than 3.5 g/day) associated with hypoalbuminemia (< 3.5 gr/dL). The nephritic syndrome was proteinuria less than 3.5 g/day associated with hematuria, hypertension (defined as an arterial blood pressure of 140/90 mm Hg or higher and/or existence of antihypertensive drug usage), and renal failure (defined as an increase of serum creatinine permanent and/or at least 50% of baseline in sequential laboratory analysis). Mixed nephrotic syndrome was defined as nephrotic syndrome coexisting with findings of HT, hematuria and renal failure. Proteinuria less than 3.5 g/day and/or isolated microscopic hematuria without clinical and laboratory signs were recorded as AUA. RPGN was defined as a doubling of serum creatinine in days and/or weeks, recorded in the hospitalization period. ‘Others’ group includes the remaining clinical presentations such as elevated serum creatinine and macroscopic or sustained hematuria without significant proteinuria. The chronic kidney disease epidemiology collaboration(CKD-EPI) equation was used to determine the estimated glomerular filtration rate^34^. The clinic(nephrology or radiology) where the kidney biopsy was performed, was also recorded.

The findings of LM were classified as ***glomerular*** lesions *sclerosis*, classified as global*(GS)* or segmental (SS) and graded 0 to 4 by percentage of involved glomeruli on biopsy as absent,  < 25%, 25–50%, 50–75%, > 75%. Cases in which segmental sclerosis were defined but the glomeruli count were not given were presented as ‘Undetermined’. *Thickened basal membrane(TBM)* and *mesengial proliferation* (*MP*), were scaled as absent, 0 or present, 1; *crescent*, graded 0 to 4 by the percentage of glomeruli as absent, < 25%, 25–50%, 50–75%, > 75%, and type of crescent as cellular, fibro-cellular and fibrous; ***tubular*** lesion, *atrophy* was recorded using the scale of 0 to 3; normal;  < 25%; 25–50%; > 50%; ***interstitial*** lesions, *inflammation*, as absent, 0 or present, 1; and *fibrosis*, like tubular atrophy); ***vascular*** lesions *vascular changing *(*VC*)*,* as absent or present of arteriosclerosis or/and arteriolosclerosis). In histopathological examination, findings of LM, thickened basal membrane (TBM), mesangial proliferation (MP), tubular atrophy (TA), interstitial fibrosis (IF) and vascular changes (VC) were evaluated. Histopathological definitions and the scorring data are tabulated and presented in Table 3Supp^35^.

Immunofluorescent microscopy was performed using fluoresceinated antibodies to immunoglobulin(Ig) IgG, IgM, IgA, C3, C4, C1q, fibrin, and both κ and λ light chains. The semi-quantitative scale of absent; ( +)mild; (+ +)moderate; (+ + +)severe was used to categorize the intensity of IFM findings. Only C4 was excluded from statistical analysis because of the insufficient count of positivity.

The study protocol was approved by ITF Clinical Research Ethics Committee, Istanbul University (no: 1131-614). In accordance with local regulations, written consent of the patients was obtained at the admission. Additional informed consent was waived by the same Ethics Committee due to the retrospective design of the study. We confirm that all resarchers were performed in accordance with relevant guidelines and all procedures performed in this study were in accordance with the Declaration of Helsinki.

The statistical evaluation were performed using the IBM SPSS Statistics for Windows, Version 26.0(IBM Corp., Armonk, NY, USA). We analyzed the normality of distribution of all parameters with Kolmogrov Smirnov test and histogram curves. The data were reported as mean value ± standard deviation(SD) for continuous numerical variables while reported as median value and percentages/inter quartiles range(IQR) for categorical ones. The possible differences between the two groups were analyzed with independent *t*-test for numerical variables if the normal distribution was determined; if not, Mann Whitney *U* test was used. One-way ANOVA or Kruskal Wallis test was used more than two groups accordingly. Crosstabulation with Chi-Square and Fischer Exact test were used in the comparison of the categorical variables. Correlation analyses were performed with Pearson’s or Spearman’s correlation tests accordingly. Statistical significance is considered when p value was detected as < 0.05.

### Supplementary Information

Supplementary Tables.

## Data Availability

We obtained the information about FSGS from the nationwide database of primary glomerular diseases that constituted by Turkish Society of Nephrology Glomerular Diseases working group (TSN-GOLD) in 2008. The can be reached at the following address: https://gold.nefroloji.org.tr/. The datasets used and/or analysed during the current study available from the corresponding author on reasonable request.
