# Supplementary material for: Clinical and histopathological characteristics of primary focal segmental glomerulosclerosis in Turkish adults
Source: Sci Rep. 2024 Mar 21;14:6748. doi: 10.1038/s41598-024-57305-6 (PMC10957996; doi:10.1038/s41598-024-57305-6)
Supplement: Supplementary file 1 — Supplementary Tables. [file 41598_2024_57305_MOESM1_ESM.docx]

**Table 1Supp:** The correlations of clinical and histopathological data, Light microscopy

| **Demographics^a^** | Glomerular lesions | | | | | | | | Tubulo-interstitial-vascular lesions | | | | | |
| --- | --- | --- | --- | --- | --- | --- | --- | --- | --- | --- | --- | --- | --- | --- |
|  | TBM | | MP | | GS | | SS | | TA | | IF | | VC | |
|  | **p** | ***r*** | **p** | ***r*** | **p** | ***r*** | **p** | ***r*** | **p** | ***r*** | **p** | ***r*** | **P** | ***r*** |
| Gender  (m=1, f=2) | NS | NS | NS | NS | NS | NS | NS | NS | * | -.080 | ** | -.105 | NS | NS |
| Hematuria | NS | NS | NS | NS | * | NS | NS | NS | NS | NS | NS | NS | NS | NS |
| Leukocyturia | NS | NS | NS | NS | NS | NS | NS | NS | NS | NS | NS | NS | NS | NS |
| Both present | NS | NS | NS | NS | NS | NS | NS | NS | NS | NS | NS | NS | NS | NS |
| Both absent | NS | NS | NS | NS | NS | NS | NS | NS | NS | NS | NS | NS | NS | NS |
| **Clinical Characteristics^b^** |  | |  | | | | | | | | | | | |
| Age, *year* | NS | NS | * | .073* | NS | NS | NS | NS | NS | NS | NS | NS | *** | .232** |
| BMI, *kg/m2* | NS | NS | NS | NS | NS | NS | NS | NS | NS | NS | NS | NS | NS | NS |
| SBP *mm Hg* | NS | NS | NS | NS | ** | .158** | NS | NS | NS | NS | NS | NS | *** | .169** |
| DBP, *mm Hg* | NS | NS | NS | NS | *** | .143** | NS | NS | NS | NS | NS | NS | ** | .098** |
| MAP, *mm Hg* | NS | NS | NS | NS | *** | .166** | NS | NS | NS | NS | NS | NS | *** | .138** |
| PP, *mm Hg* | NS | NS | NS | NS | *** | .112** | NS | NS | NS | NS | NS | NS | *** | .146** |
| **Laboratory Analysis^b^** |  | |  | | | | | | | | | | | |
| Glucose, *mg/dL* | NS | NS | NS | NS | NS | NS | NS | NS | NS | NS | NS | NS | ** | .121** |
| BUN, *mg/dL* | * | .088* | NS | NS | *** | .269** | NS | .113** | *** | .255** | *** | .265** | *** | .160** |
| Creatinine, *mg/dL* | * | .103** | NS | NS | *** | .269** | *** | .115** | *** | .338** | *** | .305** | *** | .146** |
| eGFR, *mL/min/1.73 m²* | * | -.073* | NS | NS | *** | -.406** | * | -.167** | *** | -.338** | *** | -.354** | *** | -.313** |
| Albumin, *g/dL* | NS | NS | NS | NS | *** | .164** | * | NS | *** | .114** | ** | .121** | ** | .094* |
| Cholesterol, *mg/dL* | NS | NS | NS | NS | * | .136** | ** | NS | *** | -.147** | *** | -.166** | NS | NS |
| TG, *mg/dL* | NS | NS | NS | NS | NS | NS | NS | NS | NS | NS | NS | NS | NS | NS |
| HDL *mg/dL* | NS | NS | NS | NS | ** | -.171** | NS | NS | ** | -.157** | * | -.166 | * | -.097** |
| LDL *mg/dL* | NS | NS | NS | NS | * | -.119** | NS | NS | ** | -.132** | * | -.147** | * | -.086* |
| UA, *mg/dL* | NS | NS | NS | NS | *** | .281** | NS | NS | *** | .174** | *** | .179** | *** | .127** |
| Hb, g*/dL* | NS | NS | NS | NS | NS | NS | NS | NS | .053 | -.069 | NS | NS | NS | -.076* |
| ESR, *mm/hour* | NS | NS | NS | NS | NS | NS | * | .146** | NS | NS | NS | NS | NS | NS |
| Proteinuria *(mg/day)^a^* | * | .107** | NS | NS | * | .144** | * | .109** | NS | NS | NS | NS | NS | NS |

**p*<0.05, ***p*≤0.01, ****p*≤0.001, ^a^*Chi-Square,* Mann-Whitney U, and Kruskal Wallis test, ^b^independent *t*-test, and One-way ANOVA test were used accordingly for calculation *p* values. Spearman’s rho and Pearson correlation analysis were used accordingly for *r* values. p<0.05 is considered significant. **Abbreviations:** BMI, body mass index; SBP, systolic blood pressure; DBP, diastolic blood pressure; MAP, mean arterial pressure; PP, pulse pressure; BUN, blood urea nitrogen; eGFR(estimated glomerular filtration rate); HDL, high-density lipoprotein; LDL, low-density lipoprotein; UA, uric acid; Hb, hemoglobin; ESR, Erythrocyte sedimentation rate.

**Table 2Supp:** The correlations of clinical and histopathological data, immunofluorescent microscopy

|  | Immunofluorescent staining | | | | | | | | | | | | | | | |
| --- | --- | --- | --- | --- | --- | --- | --- | --- | --- | --- | --- | --- | --- | --- | --- | --- |
|  | IgG | | IgM | | IgA | | C3 | | C1q | | kappa | | lambda | | fibrinogen | |
|  | **p** | ***r*** | **p** | ***r*** | **p** | ***r*** | **p** | ***r*** | **p** | ***r*** | **p** | ***r*** | **p** | ***r*** | **p** | ***r*** |
| **Demographics**^a^ | | | | | | | | | | | | | | | | |
| Gender(m=1, f=2) | * | -.073* | NS | NS | NS | NS | NS | NS | NS | NS | NS | NS | NS | NS | NS | NS |
| Hematuria | * | NS | * | NS | * | NS | NS | NS | * | NS | * | .085* | NS | NS | NS | NS |
| Leukocyturia | NS | NS | NS | NS | * | NS | NS | NS | NS | NS | NS | NS | * | .081* | NS | NS |
| Both present | NS | NS | NS | NS | NS | NS | NS | NS | NS | NS | NS | NS | NS | NS | NS | NS |
| Both absent | NS | NS | NS | NS | NS | NS | NS | NS | NS | NS | NS | NS | NS | NS | NS | NS |
| **Clinical Characteristics**^b^ | | | | | | | | | | | | | | | | |
| Age, *year* | NS | NS | NS | NS | NS | NS | NS | NS | NS | NS | NS | NS | NS | NS | NS | NS |
| BMI, *kg/m2* | NS | NS | NS | NS | NS | NS | NS | NS | NS | NS | NS | NS | NS | NS | NS | NS |
| SBP *mm Hg* | NS | NS | NS | NS | NS | NS | NS | NS | NS | NS | NS | NS | NS | NS | NS | NS |
| DBP, *mm Hg* | NS | NS | NS | NS | NS | NS | * | .093­* | NS | NS | NS | NS | NS | NS | NS | NS |
| MAP, *mm Hg* | NS | NS | NS | NS | NS | NS | * | .084* | NS | NS | NS | NS | NS | NS | NS | NS |
| PP,*mm Hg* | NS | NS | NS | NS | NS | NS | NS | NS | NS | NS | NS | NS | NS | NS | NS | NS |
| **Laboratory Analysis**^b^ | | | | | | | | | | | | | | | | |
| Glucose, *mg/dL* | NS | NS | NS | NS | NS | NS | NS | NS | NS | NS | NS | NS | * | .080* | NS | NS |
| BUN, *mg/dL* | NS | NS | NS | NS | NS | NS | NS | NS | NS | NS | NS | NS | NS | NS | NS | NS |
| Creatinine, *mg/dL* | NS | NS | NS | NS | NS | NS | * | NS | NS | NS | NS | NS | NS | NS | NS | NS |
| eGFR, *mL/min/1.73 m²* | NS | NS | *** | -.118** | NS | NS | NS | NS | NS | NS | NS | NS | NS | NS | NS | NS |
| Albumin, *g/dL* | NS | NS | * | -.090* | NS | NS | NS | NS | NS | NS | NS | NS | NS | NS | NS | NS |
| Cholestrol,*mg/dL* | NS | NS | * | .099** | NS | NS | NS | NS | NS | NS | NS | NS | NS | NS | NS | NS |
| TG, *mg/dL* | NS | NS | NS | NS | NS | NS | NS | NS | NS | NS | NS | NS | NS | NS | NS | NS |
| HDL *mg/dL* | NS | NS | NS | NS | * | NS | NS | NS | NS | NS | * | -.085* | NS | NS | NS | NS |
| LDL *mg/dL)* | * | .123** | * | .103 | NS | NS | NS | NS | NS | NS | * | .079 | NS | NS | NS | NS |
| UA, *mg/dl* | NS | NS | ** | .109** | NS | NS | NS | NS | NS | NS | NS | NS | NS | NS | NS | NS |
| Hemoglobin*g/dL* | NS | NS | NS | NS | NS | NS | NS | NS | NS | NS | NS | NS | NS | NS | NS | NS |
| ESR, *mm/hour* | NS | NS | NS | NS | * | NS | NS | NS | NS | NS | NS | NS | NS | NS | NS | NS |
| Proteinuria*(mg/day)^a^* | NS | NS | * | NS | * | .129** | NS | NS | NS | NS | NS | NS | NS | NS | * | .085* |

**p*<0.05, ***p*≤0.01, ****p*≤0.001, ^a^*Chi-Square,* Mann Whitney U test and Kruskal Wallis test, ^b^independent *t*-test, and One-way ANOVA test were used accordingly for calculation the *p* values. Spearman’s rho and Pearson’s correlation tests were used accordingly for *r* values. p<0.05 is considered significant. **Abbreviations:** BMI, body mass index; SBP, systolic blood pressure; DBP, diastolic blood pressure; MAP, mean arterial pressure; PP, pulse pressure; BUN, blood urea nitrogen; eGFR(estimated glomerular filtration rate); HDL, high-density lipoprotein; LDL, low-density lipoprotein; ESR, Erythrocyte sedimentation rate.

**Table 3Supp**: Definitions of histopathological findings (taken and modified from reference 35)

| Histopatholocigal finding, LM |  | Score | Definition of csore |
| --- | --- | --- | --- |
| Glomerular lesions | Sclerosis  Global, ***GS***  Fokal, ***FS*** | 0-4 | Number of glomerulosclerosis/all glomeruli   1. Absent 2. <%25 3. %25-50 4. %50-75 5. >75   Undetermined → *FS* +, but the number of glomerulus is not declared |
|  | Thickened basal membrane*,* TBM | 0,1 | 1. Absent 2. Present |
|  | Mesengial Proliferation*,* ***MP*** | 0,1 | 1. Absent 2. Present |
|  | Crescents  Cellular  Fibro-cellular  Fibrous | 0-4 | Number of glomerulosclerosis/all glomeruli   1. Absent 2. <25% 3. 25-50% 4. 50-75% 5. >75% |
| Tubulo-interstitial | Tubular atrophy, ***TA*** | 0-3 | 1. Normal 2. <25% 3. 25-50% 4. >50% |
|  | Intersititial inflamamtion | 0,1 | 1. Absent 2. Present |
|  | Interstitial fibrosis, ***IF*** | 0-3 | 1. Normal 2. <25% 3. 25-50% 4. >50% |
| Vascular | Vascular changing, ***VC*** | 0,1 | 1. Absent 2. Present |
| Histopatological findings, IFM | IgG, IgM, IgA, C3, C4, C1q, fibrin, κ and λ light chains | (+) -(++++) | (+) mild  (++) moderate  (+++) severe  (++++) very severe |

LM: Light microscopy, IFM: Immunofluorescent microscopy.
